# The Disulfiram/Copper Complex Induces Autophagic Cell Death in Colorectal Cancer by Targeting ULK1

**DOI:** 10.3389/fphar.2021.752825

**Published:** 2021-11-23

**Authors:** Yeting Hu, Yucheng Qian, Jingsun Wei, Tian Jin, Xiangxing Kong, Hongfeng Cao, Kefeng Ding

**Affiliations:** ^1^ Department of Colorectal Surgery and Oncology, Key Laboratory of Cancer Prevention and Intervention, Ministry of Education, The Second Affiliated Hospital, Zhejiang University School of Medicine, Hangzhou, China; ^2^ Zhejiang University Cancer Center, Hangzhou, China

**Keywords:** disulfiram, autophagy, ULK1, CRISPR library, colorectal cancer

## Abstract

Colorectal cancer (CRC) is highly prevalent worldwide, but there has been limited development of efficient and affordable treatment. Induced autophagy has recently been recognized as a novel therapeutic strategy in cancer treatment, and disulfiram (DSF), a well-known antialcohol drug, is also found to inhibit tumor growth in various malignancies. Recently, DSF has been reported to induce excessive autophagy in oral squamous cells; however, little is known about whether it can induce autophagy and suppress proliferation in CRC. In this study, we investigate the effect of DSF with copper (DSF/Cu) on CRC both *in vitro* and *in vivo* and find that the combination significantly inhibits CRC cell viability and mainly induces autophagy instead of apoptosis. Furthermore, we use whole genome CRISPR library screening and identify a new mechanism by which DSF triggers autophagy by ULK1. Overall, these findings provide a potential CRC treatment.

## Introduction

Colorectal cancer (CRC) ranks third in incidence and second in mortality among all cancers worldwide (Siegel et al., 2021). Because drug resistance is an important cause of death in patients with advanced CRC (Marin et al., 2012), it is urgent to design novel therapeutic strategies and identify new antitumor drugs for treatment.

Induced autophagy has recently been recognized as a novel therapeutic strategy in cancer treatment (Towers and Thorburn, 2016). Autophagy is a biological process by which large amounts of macromolecular substances and organelles are degraded in membrane vesicles (Wu et al., 2018) and is controlled by a series of precise signaling events induced by diverse signals and cellular stresses. A large number of studies demonstrates that antitumor drugs can effectively eliminate tumor cells by inducing autophagy (Towers and Thorburn, 2016). Likewise, preclinical evidence shows that inducing autophagy can help improve clinical outcomes in cancer patients. Following an initial finding by Amaravadi and colleagues (Amaravadi et al., 2007), a series of *in vitro* and *in vivo* studies confirms improved antitumor effects when diverse anticancer drugs are combined with induced autophagy. Thus, it is extremely important to explore new antitumor drugs that can induce autophagy and cause few side effects.

Disulfiram (DSF), a traditional antialcohol drug, is considered to be one of the most potent cancer chemotherapies. Although drowsiness and lethargy are common adverse effects of DSF, it can safely be used in clinical settings (Sellers et al., 1981). In 1977, Dr. Lewison reported that DSF treatment could inhibit bone metastasis in breast cancer (Lewison, 1977). Using the Danish demographic and health registries, cancer-specific mortality is elevated among previous DSF users versus those who have never used DSF; furthermore, mortality for cancers of the colon, prostate, and breast was lower among continuing users than previous DSF users (Skrott et al., 2017). Recent studies also show that DSF combined with copper (DSF/Cu) could induce NPL4 aggregation and a complex cellular phenotype, finally leading to cell death. Moreover, DSF/Cu is reported to induce significant cell cycle arrest and apoptosis, activate the stress-related ROS/MAPK and ferroptosis pathways, and inhibit the nuclear factor-*к*B (NF-*к*B) pathway in some tumor cells (Chen et al., 2006; Guo et al., 2010; Li et al., 2020). In addition, it is reported that DSF-induced autophagy in oral squamous cell carcinoma caused cell death by cristae dysfunction (Wang et al., 2021).

In this study, we assess the effect of DSF on CRC cell lines. Notably, we find that DSF/Cu can inhibit the growth of CRC cells. We further identify a novel mechanism involved in this process by which DSF/Cu eliminates CRC cells by induced autophagy, not apoptosis. More importantly, we verify the target of DSF/Cu for inducing autophagy in CRC as ULK1.

## Materials and Methods

### Cell Culture and Cytotoxicity Analysis

RKO and Ht29 cell lines were purchased from the American Type Culture Collection. DSF was procured from Macklin. In previous studies, it is demonstrated that the anticancer function of DSF is dependent on the presence of a Cu^2+^ ion (Skrott et al., 2017; Huang et al., 2021). In our study, we establish that 10 μM Cu (Copper (II) chloride dihydrate, Solarbio) is nontoxic *in vitro* and *in vivo*. *In vitro* cytotoxicity analysis was performed according to manufacturer’s instructions: RKO and Ht29 cells (7000 cells/well) were cultured overnight in 96-well plates; exposed to DSF (0.25 μM), Cu (10 μM), or a combination of both for 24, 48, and 96 h; and then analyzed for cell viability using the Cell Counting Kit-8 (KeyGEN BioTech, Nanjing, China). In parallel, CRC cells were planted into a six-well plate containing 5 ml medium at a density of 500 cells/well and exposed to DSF, Cu, or a combination of both for 96 h. Then, the cells were rinsed with 0.01% PBS, fixed with 4% paraformaldehyde for 15 min, and stained with crystal violet. The number of clones was counted using Image J software.

### Xenograft Model

The human CRC cell line RKO (1×10^6^ cells) was subcutaneously injected into Balb/c female mice (Shanghai SLAC Laboratory Animal Co., Ltd.) of body weight 15–18 g and age 6–8 weeks. After 7 days of injection, mice whose tumor size exceeded 20 mm in diameter (volume 4000 mm^3^) and developed to severely interfere with normal body functions were excluded. After the tumor grew into more than 0.1 cm^3^, the mice were randomly divided into four groups with five mice each and treated as follows (Skrott et al., 2017): 1) normal diet, 2) normal diet combined with Cu gavage (0.15 mg/kg), 3) normal diet combined with DSF gavage (50 mg/kg DSF), and 4) normal diet plus Cu and DSF gavage (50 mg/kg DSF and 0.15 mg/kg Cu). Tumor volume and weight were measured every morning.

### Whole Genome CRISPR Screening

Approximately 1×10^8^ RKO cells were infected with GeCKOv2 library A, which contains 65,386 unique sgRNA sequences targeting 19,052 genes and 1239 nontargeting controls, at a multiplicity of infection (MOI) of 0.3 to favor infection with a single viral particle/cell. Cells were puromycin-selected (5 μg/ml) for 7 days to obtain a mutant cell pool and were maintained at >500× coverage at all times after 2 days of infection.

The mutant cell pool was treated with carrier (DMSO) and DSF/Cu (0.2 µM/10 μM) for 7 and 14 days. Afterward, the cells were collected for genomic DNA extraction. The sgRNA sequence was amplified using NEBNext^®^ High-Fidelity 2X PCR Master Mix, and massively parallel amplicon sequencing was performed by Novogene Technology (Beijing, China). MAGeCK computational software was used for downstream analysis to obtain sgRNA read counts, gene rankings, and statistics.

### DSF/Cu Treatment and Sample Preparation

The concentration of DSF/Cu to kill RKO cells was determined. The stably transfected cells were exposed to 0.2 μM DSF/Cu for 5 days, and about 20% of the cells survived. Cells were cultured in a medium containing 0.1 μM DSF/Cu for 7 and 14 days, and a Blood & Cell Culture Max Kit (Qiagen) was used to extract genomic DNA, which was sequenced and analyzed by Obio Technology, China.

### qRT-PCR Assay

Total RNA from cells and xenograft tissues were isolated using TRIzol reagent (TIANGEN, Beijing, China) according to the manufacturer’s instructions. The quality and quantity of RNA was evaluated using a NanoDrop 2000 (Jiang et al., 2021) spectrophotometer (Thermo Scientific, Pittsburgh, PA, USA). cDNA was synthesized by a PrimeScript™ II 1st Strand cDNA Synthesis Kit (Takara biotechnology, Dalian, China). Quantitative real-time PCR (qRT-PCR) was performed using a standard SYBR Green PCR kit protocol (YEASEN, Shanghai, China) with a 7500 Fast Real-Time PCR System (Life Technologies, Shanghai, China). The gene expression was quantified by the Taqman probe system using the following primers and probes: ULK1 sense: 5′-GGC​AAG​TTC​GAG​TTC​TCC​CG-3′, antisense: 5′-CGA​CCT​CCA​AAT​CGT​GCT​TCT-3′; ATG16L2, sense: 5′-TTA​GCA​GCA​ACT​TAC​AAC​CAG​G-3′, antisense: 5′-ACA​CCA​CGT​CAT​TAC​AGT​AGG​A-3′; ATG12, sense: 5′-TTG​CTA​TAA​CTA​GGG​TGA​CAC​CA-3′, antisense: 5′-CCC​AAC​ACG​AAC​TGT​CTG​GA-3′; LAMP3: 5′-GCG​TCC​CTG​GCC​GTA​ATT​T-3′, antisense: 5′-TGC​TTG​CTT​AGC​TGG​TTG​CT-3′; PIK3C3: 5′-CCT​GGA​AGA​CCC​AAT​GTT​GAA​G-3′, antisense: 5′-CGG​GAC​CAT​ACA​CAT​CCC​AT-3′; GAPDH, sense: 5′-CCA​CTC​CTC​CAC​CAC​CTT​TGA​C-3′, antisense: 5′-ACC​CTG​TTG​CTG​TAG​CCA-3′. All PCR reactions were performed in triplicate, and gene expression was normalized to the expression of GAPDH.

### Western Blotting Analysis

The cells were washed twice using ice-cold PBS, harvested, and lysed with RIPA lysis buffer (#P0013D, Beyotime, Wuhan, China) supplemented with 1% cocktail (Sigma-Aldrich, Hamburg, Germany) for Western blotting analysis. The nuclear or cytoplasmic protein samples (50 μg) were electrophoresed by 12% SDS-PAGE, transferred to a PVDF membrane (Bio-Rad, USA), and stained with primary and second antibodies. Protein signals were visualized by the enhanced chemiluminescence substrate (Thermo Scientific, Pittsburgh, PA, USA) and scanned by a Tanon 5200 chemiluminescence imaging system (Tanon, Shanghai, China). The primary antibodies included rabbit anti-ULK1 antibody (1:1000, Huabio, Hangzhou, China), rabbit anti-LC3B antibody (1:1000, Cell Signal Technology, Beverly, MA, USA), rabbit anti-ATG5 polyclonal antibody (1:1000, Huabio, Hangzhou, China), rabbit anticleaved-cas3 antibody (1:1000, Huabio, Hangzhou, China), rabbit anti-PARP antibody (1:1000, Cell Signal Technology, Beverly, MA, USA), and rabbit anti-p62 antibody (1:1000, abcam, USA). The rabbit anti-GAPDH monoclonal antibody (1:5000, Cell Signal Technology, Beverly, MA, USA) was used as the loading control.

### Annexin V-FITC Apoptosis Detection

An Annexin V-FITC Apoptosis Detection Kit (Yeasen, China) was used to detect the percentage of cell death according to the manufacturer’s instructions. RKO cells were planted into a six-well plate containing 5 ml medium at a density of 5 × 105 cells/well and exposed to DSF, Cu, or a combination of both for 16 h. All the cells were harvested and resuspended in ice-cold 1× binding buffer at a concentration of 1 × 106 cells/ml. A 100-μL cell suspension was mixed with 5 μL FITC Annexin V and 5 μL PI. The mixture was incubated at room temperature in the dark for 15 min and then analyzed by a FACS Calibur flow cytometer (Beckman Coulter, CytoFLEX S).

### Immunohistochemistry and TUNEL Assay

H&E staining was performed on 4-μm paraffin sections according to standard protocol (Said et al., 2021). Tumor tissue sections were treated with xylene and gradient ethanol, and apoptosis was measured using a TUNEL Apoptosis Detection Kit (FITC) (YEASEN, Shanghai, China). Fluorescence images were acquired using Zeiss LSM 710 confocal laser microscope (Carl Zeiss, Germany) and analyzed using Zen software.

### Immunofluorescence and Electron Microscope Assay

RKO cells were planted on coverslips and treated with DSF/Cu (0.25 μM/10 μM) for 36 h. These were then fixed with 4% (w/v) paraformaldehyde in PBS for 10 min at room temperature and permeabilized with 0.5% Triton X-100 in PBS for 15 min. Cells were stained with the primary antibody, anti-LC3B antibody (1:500; Cell Signaling Technology, #3868), and incubated in blocking buffer at room temperature for 1 h or overnight at 4°C. After washing thrice with PBS containing 0.05% Tween 20, cells were incubated with the appropriate Alexa Fluor 488 and 568 secondary antibodies (Invitrogen, 1:1000) for 1 h at room temperature. The slides were washed with PBS with 0.02% Tween 20 and counterstained with DAPI (SouthernBiotech) for nuclear staining. Images were scanned with a confocal microscope (Zeiss LSM 710). Image analysis was performed by Zen software.

RKO cells were treated with DSF/Cu (0.25/10 μM) for 36 h. The culture medium was removed, and 2.5% glutaraldehyde solution was added to fix the cells for 1 h at room temperature. Cells were then harvested, washed gently thrice with PBS, and fixed in 1% osmium tetroxide–PBS for another hour. After dehydration by graded ethanol series, cells were critical point dried and sputter-coated with 10% gold. Observations were performed using a transmission electron microscope (TECNAI10, Philips, Holland).

### Establishment of ULK1 Knockdown Cell Line

The lentiviral particles containing validated short hairpin RNA directed against ULK1 are shULK1-1# sequence, 5′-CCG​GGG​TAC​CTC​CAG​AGC​AAC​ATG​ACT​CGA​GTC​ATG​TTG​CTC​TG G​AGG​TAC​CTT​TTT​G-3′ and 5′-AAT​TCA​AAA​AGG​TAC​CTC​CAG​AGC​AAC​ATG​ACT​CGA​GTC​ATG​TTG​CTC​TGG​AG G​TAC​C-3′; shULK1-2# sequence, 5′- CCG​GGC​CCT​TTG​CGT​TAT​ATT​GTA​TCT​CGA​GAT​ACA​ATA​TAA​CGC​AAA​GGG​CT T​TTT​G-3′ and 5′- AAT​TCA​AAA​AGC​CCT​TTG​CGT​TAT​ATT​GTA​TCT​CGA​GAT​ACA​ATA​TAA​CGC​AAA​GGG​C-3′; and shULK1-3# sequence, 5′- CCG​GCG​CAT​GGA​CTT​CGA​TGA​GTT​TCT​CGA​GAA​ACT​CAT​CGA​AGT​CCA​TGC​GTT​TTT​G-3′ and 5′- AAT​TCA​AAA​ACG​CAT​GGA​CTT​CGA​TGA​GTT​TCT​CGA​GAA​ACT​CAT​CGA​AGT​CCA​TGC​G-3′. The corresponding scramble control was shCtrl sequence, 5′-CGC​GTC​TAT​AAC​GGC​GCT​CGA​TAT​TTC​AAG​AGA​ATA​TCG​AGC​GCC​GTT​ATA​GTT​TTT​GGA​AAT-3′ and 5′-CGA​TTT​CCA​AAA​ACT​ATA​ACG​GCG​CTC​GAT​ATT​CTC​TTG​AAA​TAT​CGA​GCG​CCG​TTA​TAG​A-3′. These were purchased from Tsingke Biotechnology Co., Ltd., (Beijing, China). Lentiviral infections were performed according to the manufacturer’s instructions. Briefly, RKO cells were plated at 50% confluence. On the day of infection, the culture medium was replaced by complete medium with appropriate lentiviral particles (MOI = 20) and polybrene (5 μg/ml). Following 24 h of infection at 37°C, viral supernatant was replaced by fresh media. Another 48 h later, infected cells were treated with 5.0 μg/ml puromycin for 2 weeks for stable clone selection. The knockdown efficiency was determined by qRT-PCR and Western blot analysis.

### Immunohistochemical Staining

The xenograft tumors (control and DSF/Cu groups) were deparaffinized in xylene, dehydrated in graded alcohol, and cut into sections with 4-µm thickness. The sections were deparaffinized, rehydrated, treated with 3% hydrogen peroxide, and blocked with 10% goat serum at 37°C for 30 min. After washing, the sections were incubated with anti-PARP (1:100, Huabio), anticleaved-cas3 (1:100, Huabio), and anti-LC3II (1:100, Proteintech) at 4°C overnight. The sections were then washed with PBS and incubated with appropriate HRP-conjugated secondary antibodies (1:500 dilution, ThermoFisher Scientific, Waltham, MA, USA) at 37°C for 1°h. Finally, the sections were incubated with diaminobenzidine and counterstained with hematoxylin for detection.

### Statistical Analysis

All data were statistically analyzed with GraphPad Prism 6.0 and SPSS 20.0 software. A two-tailed *t*-test was utilized to analyze the difference between the two groups. Data were presented as mean ± SD or SEM. *p* values of less than .05 were considered statistically significant.

## Results

### DSF/Cu Inhibits CRC Cell Viability *in Vitro* and *in Vivo*

**FIGURE 1:**
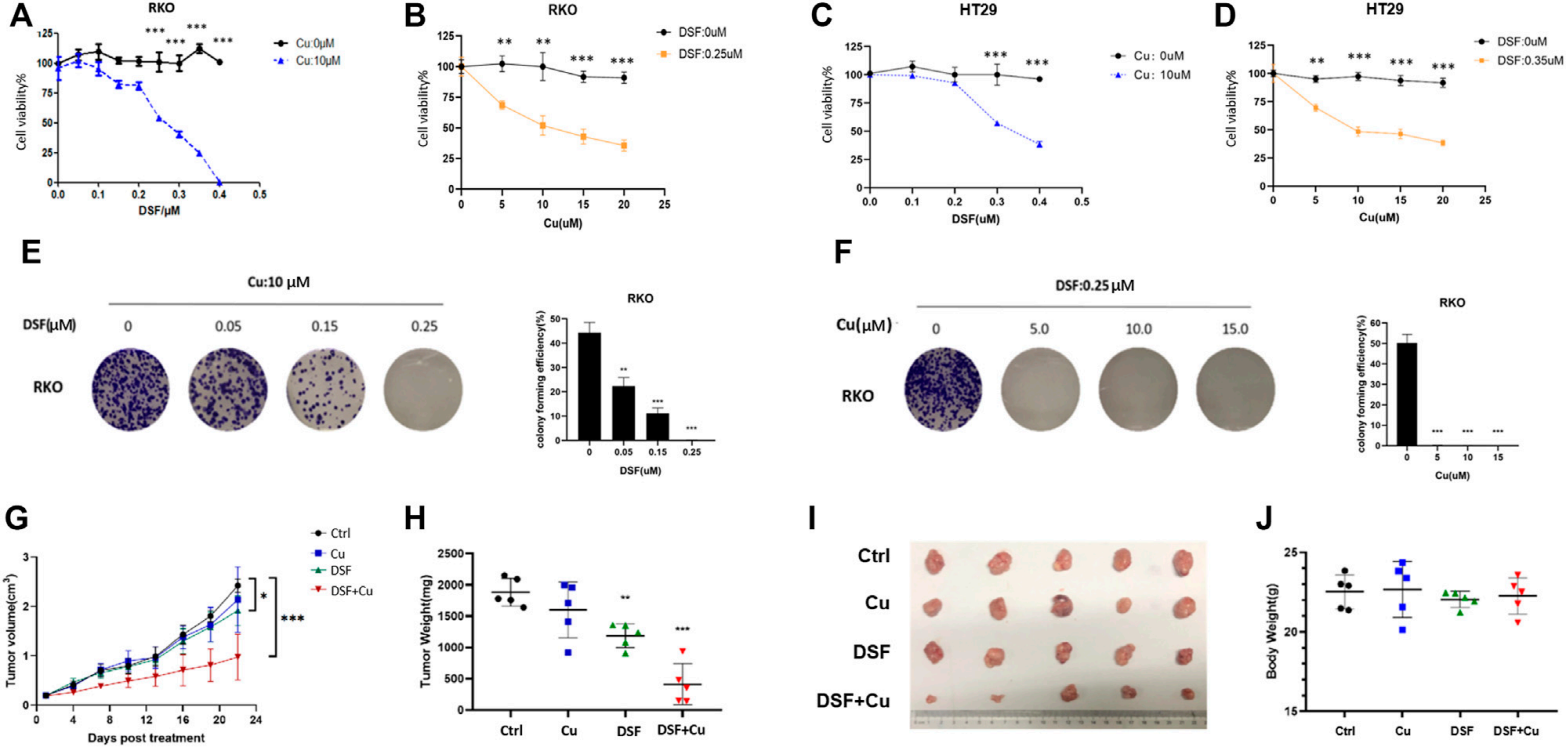
DSF/Cu inhibits CRC cell viability *in vitro* and *in vivo*. **(A**–**D)** The colorectal cancer cell lines RKO and HT29 were treated with different concentrations of DSF/Cu (DSF: 0.1–0.4 μM; Cu: 5–10 μM) for 48 h, and cell viability was evaluated by CCK8 assay. The IC50 values of DSF were 0.25 and 0.3 μM for RKO and HT29 cells, respectively. **(E)** Colorectal cancer cell line RKO was treated with different concentrations of DSF (0.05–0.25 μM) and a fixed Cu concentration (10 μM), after which inhibition effects were determined by colony formation assay. **(F)** Colorectal cancer cell line RKO was treated with different concentrations of Cu (5–15 μM) and a fixed DSF concentration (0.25 μM) after which inhibition effects were determined by colony formation assay. **(G)** The growth curves of tumors in mice treated by the indicated drugs. **(H)** Xenografts extracted from mice at day 22. The tumors by the data are shown as means ± SD. ***p < .001, *n* = 5. **(I)** Photographs of subcutaneously growing xenografts extracted from mice at day 22. **(J)** The weight of mice at day 22. Data are indicated drugs. Data are shown as means ± SD. ***p < .001, *n* = 5.

Previous studies show that DSF/Cu exerts anticancer effects in malignant tumors (Iljin et al., 2009; Nechushtan et al., 2015). To test this on CRC, we employed three individual experiments. A CCK-8 assay was used to detect the cell viability of CRCcell lines (HT29 and RKO) treated with different concentrations of DSF/Cu (DSF: 0.1–0.4 μM; Cu: 5–10 μM). The results show that DSF/Cu inhibits CRC cell viability in a DSF dose-dependent manner (Figure 1A–D). The colony formation ability of RKO treated with different concentration of DSF/Cu for 9 days was detected, and results show that the number of colonies was reduced by more than 50% in the group treated with DSF/Cu (0.25/10 μM) compared with the DMSO group (Figure 1E,F). To investigate the anticancer effect of DSF/Cu *in vivo*, the subcutaneous xenograft models were employed. We found that the tumor volumes of xenografts treated for 22 days by intragastric administration of DSF/Cu (50 mg/kg DSF, 0.15 mg/kg copper gluconate) were significantly smaller than if treated with the vehicle. The average tumor volumes were 50% smaller than that of the control group (Figure 1G). Further, the average weight of the tumor tissue within the group was reduced by more than 75% (Figure 1H,I). Meanwhile, the body weights of mice were stable in both groups (Figure 1J). These results show that DSF/Cu inhibited CRC cell viability both *in vitro* and *in vivo*.

### DSF/Cu inhibits CRC cell viability through an apoptosis-independent pathway.

**FIGURE 2:**
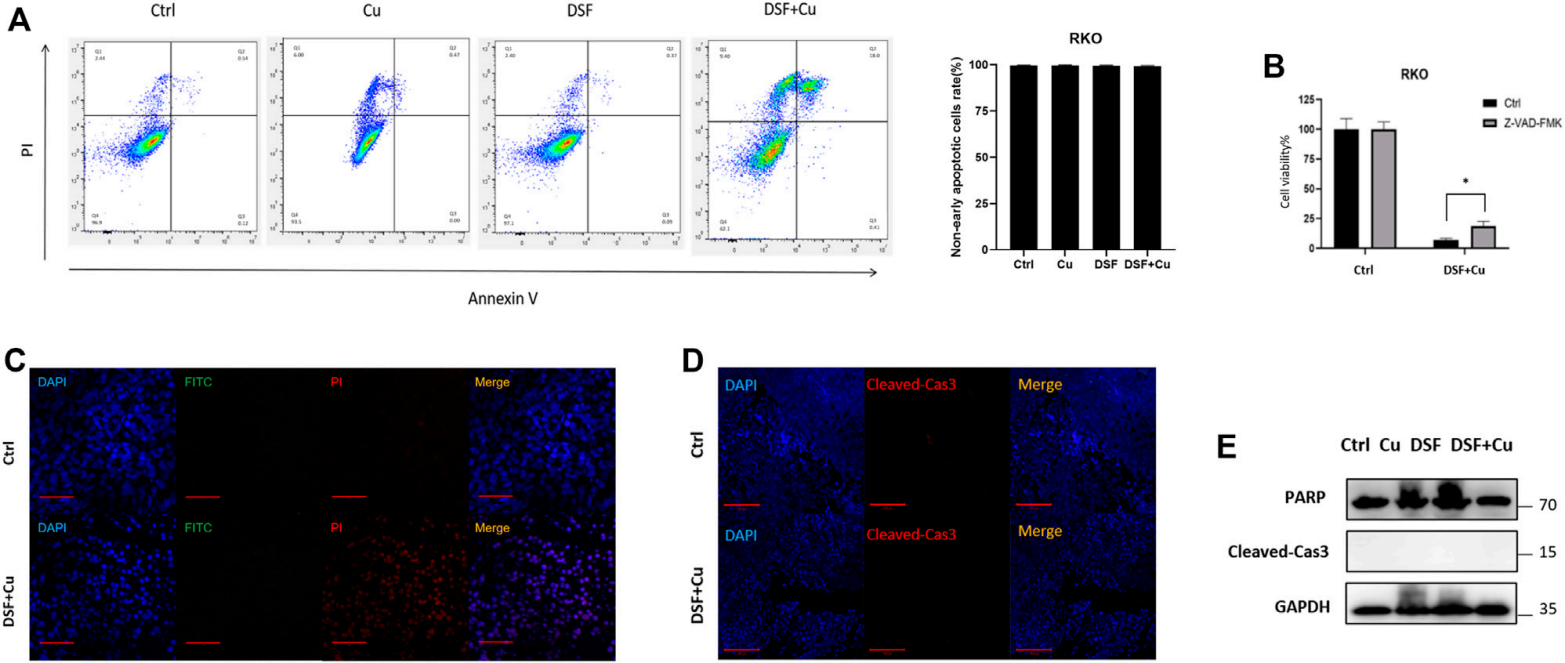
DSF/Cu inhibits CRC cell viability through an apoptosis-independent pathway. **(A)** Flow cytometry with Annexin V/PI staining proved that DSF/Cu could not increase Annexin V+/PI + cells. Data are shown as means ± SD. *p* > .05, *n* = 3. **(B)** Apoptosis inhibitor Z-VAD-FMK could partially reverse the inhibitory effect caused by DSF/Cu (0.25/10 μM). **(C)** TUNEL assay showed no significant different DNA damage between control and DSF/Cu groups. **(D,E)** Xenograft tumor tissues were examined the expression of PARP and cleaved-Cas3 by immunofluorescence and Western blot.

Recently, it was reported that DSF/Cu inhibits the viability of HCT116 and LoVo cells through apoptosis (Huang et al., 2021). Hence, we checked whether DSF/Cu induced the apoptosis of HT29 and RKO. An annexin V apoptosis assay was employed to test the apoptosis induced, and we found that the proportion of early apoptotic cells account for 2% (Figure 2A) while nearly 18% leans toward necrosis. We used an apoptosis inhibitor Z-VAD-FMK to reduce the apoptosis in RKO treated with DSF/Cu, but only a small ratio of cell viability could be reversed by this (Figure 2B). Furthermore, a TUNEL assay was used to detect the apoptotic cells in xenograft tumor tissues, and few apoptotic cells were found (Figure 2C). Similar to the TUNEL assay results, xenograft tumor tissues were examined by immunofluorescence and Western blot, and we found that there were no differential levels of cleaved-caspase3 and PARP with DSF/Cu treatment as compared with the control group (Figures 2D,E). These results indicate that DSF/Cu inhibits CRC cell viability in an apoptosis-independent pathway.

### DSF/Cu Inhibits CRC Cell Viability by Inducing Autophagy as Revealed by Crispr-cas9 Library Screening

**FIGURE 3:**
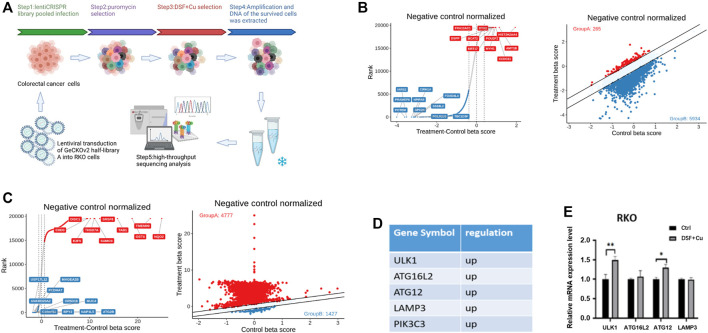
DSF/Cu inhibits CRC cell viability by inducing autophagy as revealed by CRISPR-Cas9 library screening. **(A)** Schematic diagram illustrating the workflow for genome-wide CRISPR-Cas9 knockout library screening (CRISPR: Clustered Regularly Interspaced Short Palindromic Repeats). **(B,C)** The distribution display of beta value of different genes in day 0 group and DSF treatment group for 7 **(B)** or 14 **(C)** days. Red points represent positive screening genes, and the blue ones are negative screening genes, **(D)** The positive screening genes related to autophagy. **(E)** qRT-PCR verified the expression of the upregulation autophagy-related genes in the control group and DSF/Cu. *p < .05; **p < .01; ***p < .001.

To identify the apoptosis-independent mechanism of DSF/Cu that inhibits cell viability of CRC cells, we utilized a library of GeCKOv2 CRISPR library A, which contains 65,386 unique sgRNAs targeting 19,052 protein-coding genes, and 1864 microRNAs. The human GeCKOv2 CRISPR library A is used to generate mutant cell banks. After 14 days of puromycin selection, the infected cells were inoculated into separate dishes and treated with DSF/Cu for 7 and 14 days (Figure 3A). As a result, 85% of the cells died, and the DNA of the surviving cells was extracted for PCR and NGS analysis. The genes were sorted based on the number of sgRNAs and NGS readings. As shown in the scatterplot of the number of sgRNAs and the corresponding gene sequencing reads, the distribution of the detected genes in each sgRNA shows the upregulated and downregulated genes in the 7- and 14-day samples (Figure 3B,C). We used negative selection to identify genes by which DSF/Cu inhibits in CRC cells. From NGS analysis, we identified five target genes (ULK1, ATG16L2, ATG12, LAMP3, PIK3C3) (Figure 3D) and, among the five genes, verified the expression changes of these five autophagy-related genes in RKO cell lines by qRT-PCR and found that ULK1 was significantly upregulated in both cell lines (Figure 3E). These results suggest that DSF/Cu may induce autophagy of CRC.

**FIGURE 4:**
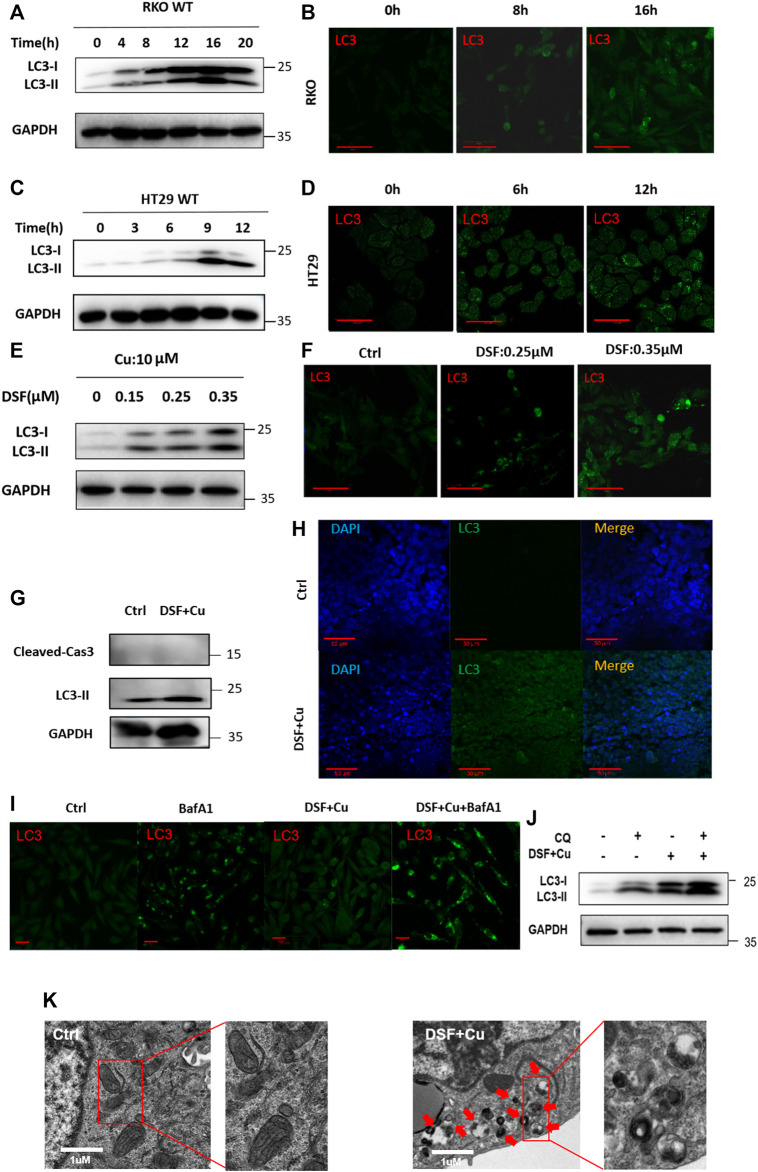
DSF/Cu activates autophagy flux. **(A, C)** RKO and HT29 cells treated with DSF/Cu (0.25/10 μM) for 0–16 h were analyzed by Western blot. **(B, D)** RKO and HT29 were treated with DSF/Cu (0.25/10 μM) for 0–16 h. The distribution of LC3 was examined by immunofluorescence. **(E–F)** The expression of LC3II was detected by Western blotting and immunofluorescence in RKO after incubation with different concentrations of DSF (0–0.35 μM) and fixed Cu (10 μM) for 12 h **(G,H)** The expression of LC3 was detected by Western blotting and immunofluorescence in xenografts. **(I)** The expression of LC3 was detected by immunofluorescence in RKO treating with DSF/Cu (0.25/10 μM) for 12 h in the presence or absence of BafA1 (0.5 μM). **(J)** The expression of LC3 was detected by Western blot in RKO treating with DSF/Cu (0.25/10 μM) for 12 h in the presence or absence of CQ (5 μM). **(K)** Representative images of autophagosome in RKO after incubation with DSF/Cu (0.25/10 μM) for 36 h. The red arrow indicates autophagosome structure. Scale bars: 1 μm.

To verify this further, we examined the characteristics of autophagy in RKO and HT29 cell lines treated with DSF/Cu. We found that DSF can effectively induce the expression of LC3II in a dose- and time-dependent manner (Figure 4A–H). Autophagy flux analysis also showed that DSF/Cu can further enhance the accumulation of LC3-II induced by CQ or bafilomycin A1 (BafA1). We inhibited autophagy using CQ (5 μM) an hour before treating RKO cells with DSF/Cu (0.25/10 μM) and analyzed the expression level of autophagy using Western blot. Autophagic flux increased following DSF/Cu treatment with BafA1 (Figure 4I) and CQ pretreatment was also found to enhance autophagy (Figure 4J). The autophagosome was confirmed by transmission electron microscopy (Figure 4K). Immunohistochemistry in xenograft tumor tissues showed that LC3 was significantly enhanced in the DSF/Cu group compared with the control group, and the expression of Caspase-3 and PARP had no change (Supplementary Figure S1). These results indicate that DSF/Cu in CRC cells significantly induced autophagy.

### DSF/Cu Induces autophagic Cell Death by Upregulating ULK1

**FIGURE 5:**
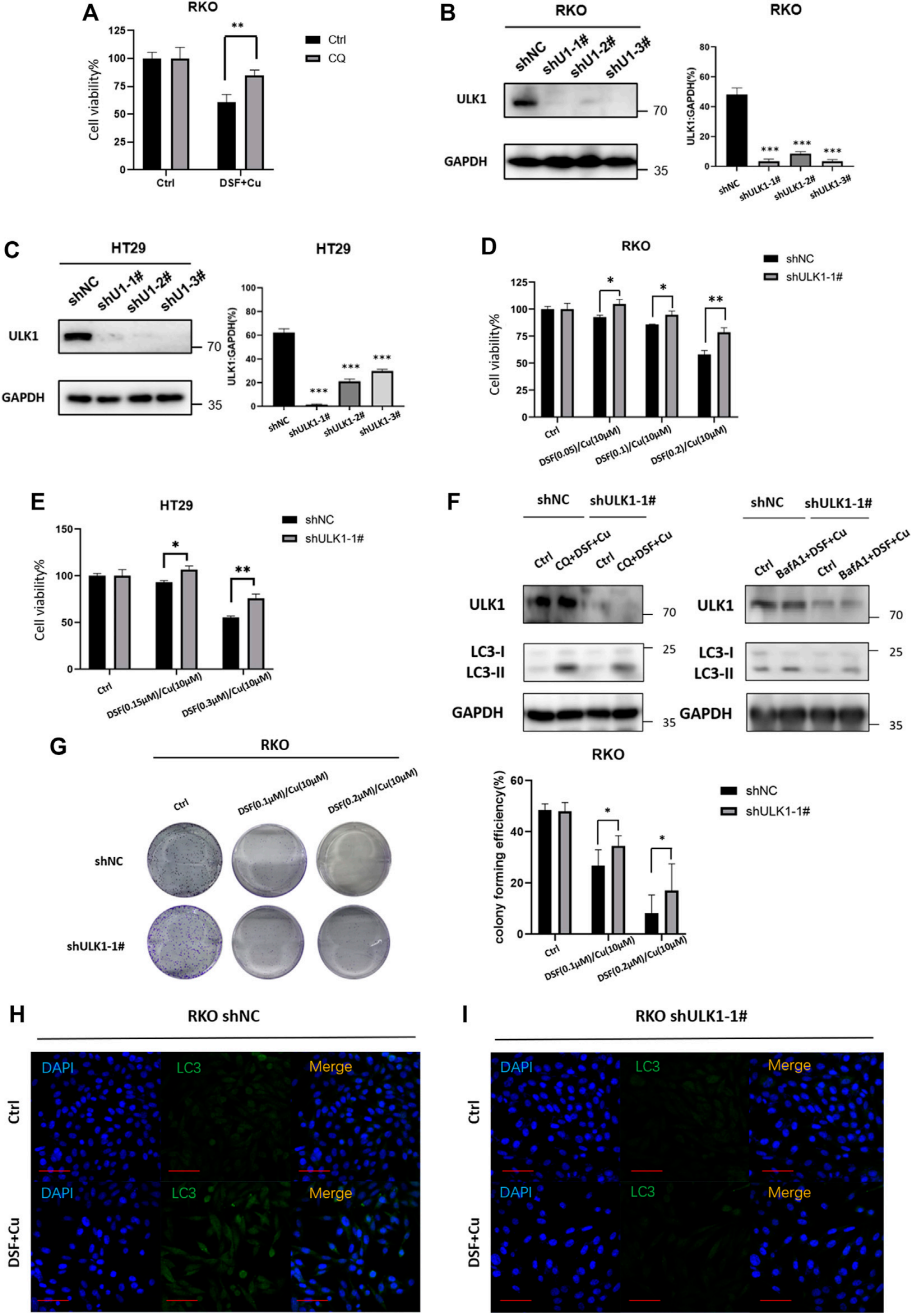
DSF/Cu induced autophagic cell death by upregulating ULK1. **(A)** CQ can partially reverse the cell viability caused by the DSF/Cu treatment in RKO (*p* < .01). **(B,C)** Low expression of ULK1 in CRC cell lines is established in RKO and Ht2, and the efficiency was verified by Western blot and qRT-PCR. **(D,E)** CCK8 assay showed that cell viability could be partially reversed in the low ULK1 expression group after incubation with DSF/Cu (DSF 0–0.2 μM/Cu 10 μM in RKO cells, DSF 0–0.3 μM/Cu 10 μM in HT29 cells) for 48 h (*p* < .01). **(F)** Western blot showed that DSF/Cu combined with CQ or Bafa1 could not enhance the accumulation of LC3-II in shULK1-RKO. **(G)**Colony formation assay showed that cell viability could be partially reversed in the low ULK1 expression group after incubation with DSF/Cu (0.25/10 μM) for 48 h (*p* < .01). **(H,I)** The expression of LC3 detected by immunofluorescence in the shULK1-RKO group was lower than the shNC-RKO group after incubation with DSF/Cu (0.25/10 μM) for 48 h (*p* < .01).

We next verified whether DSF/Cu may affect the growth of cancer cells due to its autophagy activation. Autophagy inhibitor CQ (5 μM, 2 h) can partially inhibit the cytotoxicity of DSF/Cu (Figure 5A), indicating that DSF/Cu can induce autophagic cell death in CRC cells, but inhibiting autophagy cannot completely reverse the cytotoxicity caused. To find specific targets, we use shRNA to interfere with autophagy-related genes and transduce RKO and HT29 cell lines, verifying the reduced ULK1 expression by qPCR and Western blotting. More than 90% of ULK1 expression was reduced in shULK1 cells compared with the control groups (Figure 5B,C). We next examined the effect of downregulation of ULK1 on cell growth. We observed a significant growth increase in the RKO-shULK1 group (*p* < .05) (Figure 5D,E). Similarly, the number of cell colonies of shULK1 was also more than that of the control group (*p* < .01) (Figure 5G). Autophagy flux analysis also showed that DSF/Cu combining with CQ or BafA1 could not enhance the accumulation of LC3-II in shULK1-RKO compared with shNC-RKO(Figure 5F). Furthermore, knockdown of ULK1 could significantly suppress the expression of LC3 after DSF/Cu treatment in RKO as verified by immunofluorescence (Figures 5H,I). Moreover, the expression of LC3 and ULK1 were increased in the xenograft tumor tissues treated with DSF/Cu group by Western blot (Supplementary Figure S2). These results suggest that DSF/Cu induces autophagic cell death by upregulating ULK1.

## Discussion

In this study, we find that DSF/Cu could inhibit proliferation of CRC both *in vitro* and *in vivo*. More importantly, it can induce the autophagy of CRC instead of apoptosis; inducing autophagy is a novel strategy in cancer treatment. Furthermore, we identify a new mechanism by which DSF triggers autophagy by ULK1. These findings provide a novel colorectal cancer treatment.

DSF is a traditional antialcoholism drug that has been used for more than 60 years (Iljin et al., 2009) and is found to have antitumor effects in various tumors (Cen et al., 2004; Chen et al., 2006; Skrott et al., 2017). Drowsiness and lethargy are the common adverse effect in treating with DSF, and it can safely be used in the clinic (Sellers et al., 1981). A previous IIb clinical trial shows that the combination of DSF with chemotherapy treatment was well tolerated and appeared to prolong survival in nonsmall cell lung cancer patients. (Nechushtan et al., 2015; Wu et al., 2018). Huang and his colleagues conducted a phase II study aimed to estimate the potential effectiveness of DSF/Cu to resensitize recurrent GBM to TMZ (Huang et al., 2019). The key mechanism of the antitumor role played by DSF is that it can combine with copper to form complexes (Guo et al., 2010; Skrott et al., 2017); DSF/Cu incidentally induces apoptotic cell death in breast cancer *via* inhibition of the proteasome activity (Chen et al., 2006). Moreover, DSF/Cu could intensively impair mitochondrial homeostasis, enhance lipid peroxidation, and eventually result in ferroptotic cell death in hepatocellular carcinoma (Ren et al., 2021). In our study, we further confirm the Cu-dependent cytotoxic effect of DSF on CRC cells. Previous research indicates that DSF/Cu achieves the purpose of an antitumor effect by inducing apoptosis (Cen et al., 2004; Tardito et al., 2011; Yip et al., 2011). However, our study shows that this is not the main mechanism by which DSF/Cu inhibits proliferation in CRC. Through the Annexin V apoptosis assay, only 2% of early apoptotic cells were induced by DSF/Cu although about 18% of necrosis was found in the DSF/Cu group. Moreover, only a small ratio of cell viability inhibited by DSF/Cu could be reversed by the apoptosis inhibitor Z-VAD-FMK, which indicates that the apoptosis pathway is not vital. To determine further the mechanism caused by DSF/Cu treatment, we find that the key genes changed by the DSF/Cu focused on the autophagy pathway by using the CRISPR-Cas9 screening system.

Autophagy is widely associated with tumor-suppressive mechanisms as it causes genomic instability, tumorigenesis, and malignant transformation (Udristioiu and Nica-Badea, 2019; Xia et al., 2021). Although autophagy was discovered more than 50 years ago, its role in cell growth and death is still controversial (Xia et al., 2021). In cancer cells, autophagy might contribute to cell death *via* autophagic cell death or apoptosis. Our observations, for the first time, demonstrate that DSF/Cu induces CRC cell autophagy by regulating Unc-51–like autophagy activating kinase 1(ULK1).

ULK1 is a mediated complex (ULK1/2, ATG13, ATG101, and FIP200 proteins) and is an initiator for pre-autophagosomal structure. It is activated by 5′ AMP–activated protein kinase and can initiate autophagosome biogenesis under periods of nutrient stress by directly activating many components of the autophagic machinery (Kim et al., 2011). In this study, the knockdown of ULK1 suppressed autophagy and reversed cell viability as DSF/Cu did. The effects of DSF/Cu were abolished by the restoration of ULK1. Although it has been reported that autophagy is involved in the regulation of cell viability, we are the first to report that DSF/Cu regulates autophagy to inhibit proliferation in CRC through ULK1 pathway.

## Conclusion

Our study reveals that DSF/Cu regulates autophagy to inhibit proliferation in colorectal cancer through the ULK1 pathway, which provides new evidence for employing this existing drug toward a novel anticancer use.

## Data Availability

The original contributions presented in the study are included in the article/Supplementary Material, further inquiries can be directed to the corresponding author.
