# Supplementary figures and images for: The Disulfiram/Copper Complex Induces Autophagic Cell Death in Colorectal Cancer by Targeting ULK1
Source: Front Pharmacol. 2021 Nov 23;12:752825. doi: 10.3389/fphar.2021.752825 (PMC8650091; doi:10.3389/fphar.2021.752825)

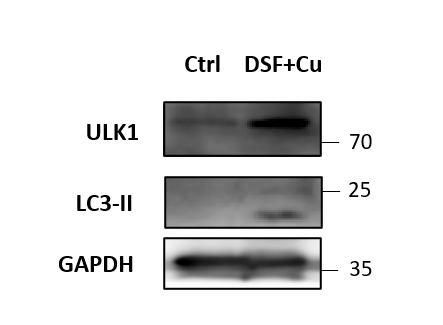

Supplement: Supplementary file 1 [file Image2.TIF]

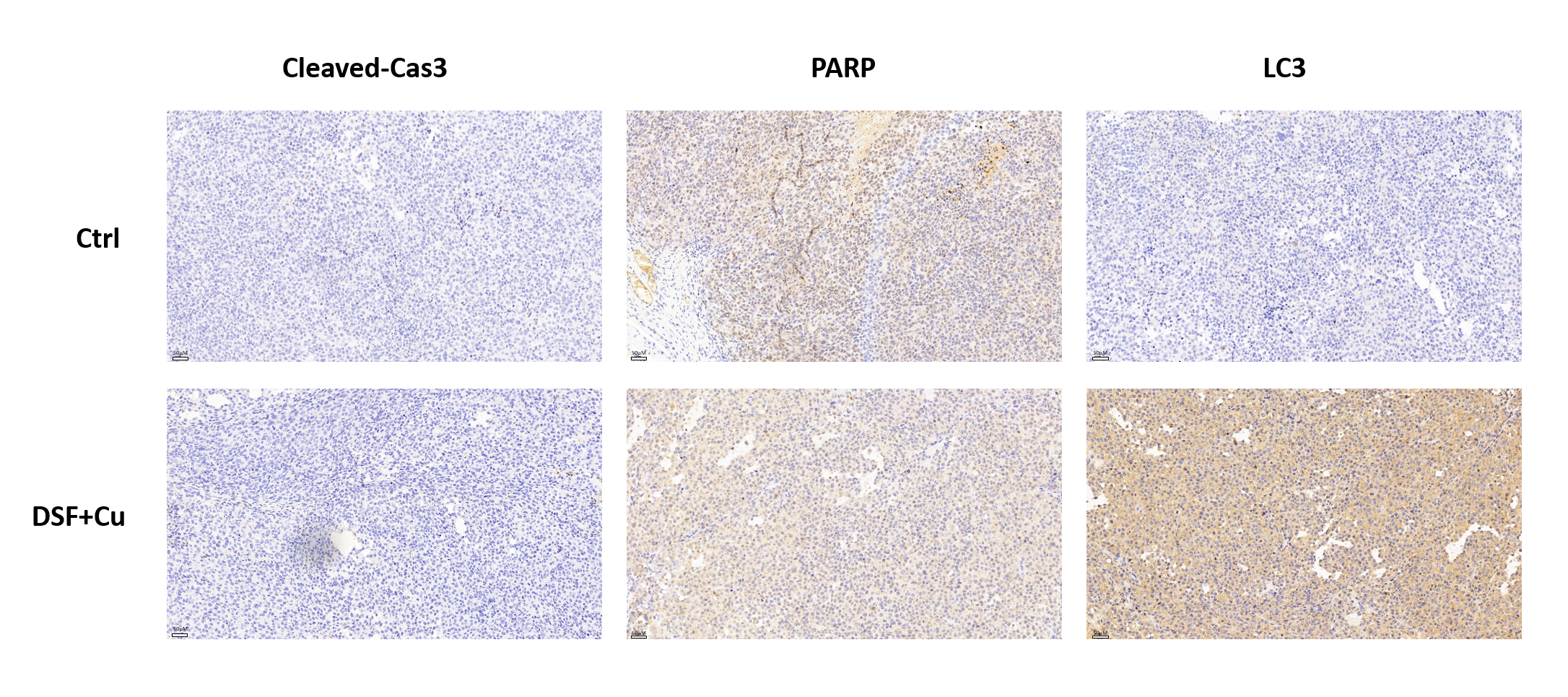

Supplement: Supplementary file 2 [file Image1.TIF]
