# Supplementary material for: The Disulfiram/Copper Complex Induces Autophagic Cell Death in Colorectal Cancer by Targeting ULK1
Source: Front Pharmacol. 2021 Nov 23;12:752825. doi: 10.3389/fphar.2021.752825 (PMC8650091; doi:10.3389/fphar.2021.752825)
Supplement: Supplementary file 3 [file DataSheet1.docx]

Supplementary figure legends

Figure S1. Immunohistochemistry in xenograft tumor tissues. LC3 was significantly enhanced in the DSF/Cu group compared with control group , while the expression of Caspase-3 and PARP had no changes.

Figure S 2. The expression of LC3 and ULK1 were increased in the xenograft tumor tissues treated with DSF/Cu group by western blot.
